# Back-on-Track: Protocol for Randomised Controlled Feasibility Trial of Behavioural Activation in Farmers with Mood Problems

**DOI:** 10.3390/ijerph23020199

**Published:** 2026-02-03

**Authors:** Alison Kennedy, Richard Gray, Martin Jones, Anna Greene, Lauren Mitchell, Meera Senthuren, Suzy Malseed, Feby Savira, Kelly Barnes, Kate Gunn, Susan Brumby

**Affiliations:** 1 National Centre for Farmer Health, School of Medicine, Deakin University, Tyers Street, Hamilton 3300, Australia; suzymalseed@gmail.com (S.M.); feby.savira@deakin.edu.au (F.S.); susan.brumby@wdhs.net (S.B.); 2 School of Nursing and Midwifery, La Trobe University, Melbourne 3096, Australia; 3 School of Nursing and Midwifry, Edith Cowan University, Perth 6027, Australia; martin.jones@ecu.edu.au; 4 National Centre for Farmer Health, Western District Health Service, Hamilton 3300, Australia; agreene@wdhs.net (A.G.); kjbarnes@wdhs.net (K.B.); 5 Clinical Trials Platform, La Trobe University, Melbourne 3096, Australia; l.mitchell4@latrobe.edu.au (L.M.); m.senthuren@latrobe.edu.au (M.S.); 6 IIMPACT in Health, Department of Rural Health, Allied Health and Human Performance, University of South Australia, Adelaide 5001, Australia; kate.gunn@unisa.edu.au

**Keywords:** farmer mental health, rural health, behavioural activation, peer-delivered, depression, feasibility trial, randomised clinical trial, peer worker, co-design

## Abstract

The mental health of people living in farming communities has been identified as an important public health issue. Cumulative exposure to a range of situational factors contributes to heightened risk of poor mental health and suicide. Access to evidence-based psychological treatments is limited by the availability of skilled mental health professionals. The aim of this trial—co-designed by members of the farming community—is to establish the feasibility of conducting randomised controlled, trial-testing, peer-worker-delivered Behavioural Activation in the farming community. We will undertake a single-blind, parallel group, randomised controlled feasibility trial in rural Australia. People living in farming communities aged over 15 years and experiencing moderate to moderately severe depression symptoms will be included in the trial. Participants will be randomly allocated on a 1:1 ratio to 10 sessions of peer-worker-delivered behavioural activation (Back-on-Track) or a self-help workbook (Managing Stress on the Farm). Peer workers are members of the farming community that have completed training in behavioural activation and demonstrated competence. Feasibility outcomes include establishing recruitment rates, willingness to be randomised, dropout rate from trial, acceptability of peer delivered behavioural activation, and willingness to complete trial measures. The trial will contribute high quality evidence of the feasibility of undertaking a full-scale, randomised controlled trial of peer-delivered Behavioural Activation in farming communities in rural Australia.

## 1. Background and Rationale

Suicide risk in farming populations is up to twice that of the general Australian population [1,2,3]. Although higher rates of diagnosed mental illness have not been clearly identified in this population [4], the association between mood and suicidal thinking seems to differ between members of the farming community and the general population. There is strong evidence for a range of situational factors (influenced by the farming context) that negatively influence farmer mental health and suicide risk [5,6,7,8,9,10].

There is some evidence that farmers are generous at providing help to others but are reluctant to ask for help themselves [11]. Most farmers live in communities in which accessing specialist mental health services is challenging [12]. Where support is available, health services and health care workers may not understand the realities of life and work in the farming environment [13]. The unique barriers to support seeking identified in farming communities may deter people from seeking help for mood problems, particularly when they have had previous negative experiences [14]. However, finding health care professionals with an understanding of farming practices and community values has been identified as an important facilitator to help seeking by Hull et al. [15].

There is sound evidence from systematic reviews of randomised controlled trials that Behavioural Activation is a safe and effective treatment for depression—a predictor of suicide [16,17]. A Cochrane review included 53 studies involving 5495 participants and concluded that there was evidence of moderate certainty that Behavioural Activation was more effective than treatment as usual [17].

Behavioural Activation is a brief psychological treatment focused on increasing behaviours that enhance mood and reducing avoidance behaviours (e.g., sitting alone and ruminating) that negatively impact mood. Unlike Cognitive Behavioural Therapy (CBT), workers without specialist mental health training can learn to deliver Behavioural Activation with minimal (typically described as around 5 days) training and ongoing supervision and support [16]. Consequently, Behavioural Activation could be delivered at-scale to people experiencing depression or low mood in communities where access to specialist mental health services is limited, such as farming communities in rural Australia.

There is growing evidence that peer-delivered mental health care services reduce relapse and rehospitalisation, and improve empowerment, hope, self-efficacy, engagement, and recovery [18]. Working with members of the farming community to deliver Behavioural Activation to their peers (enabling the shared context, characteristics, and cultural awareness to ‘walk in their shoes’) has the potential to overcome many well-established barriers to mental health help seeking and improve outcomes for this at-risk group [19].

### 1.1. Objectives

The objectives of this feasibility trial address (i) the recruitment, training, and retention of peer workers, (ii) the recruitment, randomisation, and completion of intervention and outcome measures by members of the farming community with depression, and (iii) fidelity of intervention delivery. Trial feasibility will be established if the following outcomes are met:

- Peer workers
  - Ten peer workers are recruited to participate in the trial.
  - Eight peer workers successfully complete the Behavioural Activation training package.
  - Six peer workers deliver the Back-on-Track intervention to at least one participant.
  - Five peer workers are retained through to the completion of the trial.
  - Peer workers attend at least eight of the 13 group supervision sessions.
- Members of the farming community with depression (participants)
  - Approximately 200 indicated that they are interested in taking part in the research.
  - Forty participants meet eligibility criteria and consent to participate in the trial.
  - Forty participants complete baseline (week 0) measures.
  - Forty participants agree to be randomised.
  - Forty participants start the trial.
  - Sixteen participants complete the experimental intervention (defined as attending at least six of the 10 Behavioural Activation sessions). Note: there is no feasibility measure for level of completion for the control group given provision of treatment as usual (Managing Stress on the Farm).
  - Thirty-two participants complete week 10 measures.
  - Twenty-eight participants complete week 26 measures.
- Ninety percent of the Behavioural Activation sessions are delivered to an at least satisfactory level of fidelity to model (independently determined using the Behavioural Activation fidelity assessment) [20].

The trial is not powered or designed to detect statistical between-groups differences in primary or secondary outcomes, and no statistical analysis will be undertaken or reported.

### 1.2. Public Involvement Statement

The feasibility trial was co-designed with people living and working in farming communities, as reported previously [19]. Peer worker training and trial design were heavily influenced by feedback from the co-design process and ongoing community consultation. For example, local strategies for recruitment of peer workers and trial participants were informed by a Community Reference Group. Lived experience researchers are also part of the investigator team and have decision-making authority over the conduct of the trial.

## 2. Methods

### 2.1. Trial Design

A single-blind (researcher), parallel group, 1:1 allocation, randomised controlled feasibility trial using the Australian Teletrial Program [21] teletrial model—a group of study sites working together to conduct a clinical trial.

### 2.2. Trial Setting

Based on Behavioural Activation, Back-on-Track is a community-based intervention that is delivered by peer workers in a participant’s home, their workplace (e.g., the farm), a mutually agreed public location, or via online video call (Zoom or the healthdirect [22] Video Call service). Participants will be recruited from three dairy farming regions in rural Victoria, Australia. Whilst the trial is located in dairy farming regions, participants do not need to be dairy farmers per se.

### 2.3. Eligibility Criteria (Peer Workers)

People aged over 18 years with lived experience living or working in a rural farming community will be eligible to apply to be peer workers. Peer workers may have personal experience of mental stress or distress, or previous mental ill-health. However, this is not an inclusion criterion for this trial.

### 2.4. Recruitment (Peer Workers)

Peer workers will be recruited by the following means:

(i) Inviting expressions of interest from people living in the local communities we are seeking to recruit from (e.g., advertising via local industry newsletters, social media, and flyers).
(ii) Direct approach from Community Reference Group members in the local communities we are recruiting from.
(iii) Advertisement of peer worker positions more broadly through National Centre for Farmer Health networks, including social media and monthly newsletters.

Peer workers will be employed by Western District Health Service, which provides primary, secondary, and tertiary health care in rural Australia. They will be employed on a casual contract as complementary therapy workers and will complete standard requirements of employment by Western District Health Service (e.g., pre-employment interview, reference checks, pre-employment medical check, police check, Working with Children Check, workplace competencies). Peer workers will also be required to complete a Participant Information and Consent Form (PICF).

All employment costs associated with the trial (training, police check, working with children check, travel) will be reimbursed through the project grant.

### 2.5. Eligibility Criteria (Participants)

Forty farming community members (participants) with depression will be recruited.

#### 2.5.1. Inclusion Criteria

1. Over 15 years of age.
2. A Patient Health Questionnaire (PHQ-9) score of between five and 19.
3. A member of the farming community (farm owners and managers, farm workers, members of farming families, members of agriculture-dependent communities) within geographic proximity of the three target farming regions.

#### 2.5.2. Exclusion Criteria

1. Commenced any medication for the treatment of depression in the past 4 weeks.
2. Current psychological (e.g., Cognitive Behavioural Therapy) treatment for a mental health condition.
3. Previous treatment with Transcranial Magnetic Stimulation or Electroconvulsive Therapy for depression.
4. Medically confirmed diagnosis of psychosis, personality disorder, or cognitive impairment.
5. Serious long-term health condition that may necessitate hospital admission or regular contact with secondary health services during the trial.
6. Active suicidality or report of a suicide attempt in the past 2 months.

### 2.6. Interventions

#### 2.6.1. Experimental (Back-on-Track)

Back-on-Track is a 10-session psychological intervention based on Behavioural Activation. The intervention is focused on initiating and/or reinstating activities which are meaningful to them by scheduling activities, monitoring behaviours, and identifying where changing these behaviours and activities may be beneficial to improving their mood. Throughout the intervention, participants will be asked to complete worksheets and engage in discussion with their peer workers to assist with monitoring these activities and behaviours.

##### Peer Worker Training

Peer workers will complete the University of South Australia Professional Certificate in Behavioural Activation for Depression [23]. This course will enable peer workers to practice key Behavioural Activation skills, including the following:

- Teaching people to acquire the skills to monitor their mood.
- Helping individuals recognise the relationship between behaviours and their impact on mood.
- Supporting individuals to develop skills to minimise avoidance and rumination behaviours that may negatively impact mood.
- Assisting people to schedule behaviours/activities that may enhance mood.

Behavioural Activation training typically takes around 56 h to complete, divided into a series of four modules that can be completed over a 3-month period. Whilst a substantial commitment for trainees, we have calibrated that this is the required amount of time necessary to ensure peer workers are able to deliver Behavioural Activation with a high degree of fidelity to model.

We anticipate that by utilising an asynchronous Behavioural Activation platform, this will enable us to provide training at-scale to peer workers within the context of a definitive trial and subsequent implementation strategy.

Peer worker competency to deliver Behavioural Activation will be demonstrated through expert review of three video recordings demonstrating the application of Behavioural Activation by peer workers in a simulated session. Each recording will focus on different Behavioural Activation skills, including mood monitoring, recognising behavioural patterns, and activity scheduling.

Peer workers will receive a further 20 h of skills training focused on the following:

- Rationale for peer delivered interventions.
- Overview of depression and treatments, including differences in presentations between young people and adults.
- Effective communication.
- Confidentiality.
- Working safely with people at risk of suicide.
- Peer worker self-care and safety.
- Dealing with difficult situations.
- Conflicts of interest.
- Referral pathways for participants at risk.

The full training package takes approximately 76 h to complete and peer workers will need to pass the standardised competency assessment to successfully complete the training. During delivery of the intervention, peer workers will receive fortnightly online group supervision with a clinical psychologist and Behavioural Activation specialist to support them whilst working with participants.

#### 2.6.2. Control (Managing Stress on the Farm)

Participants allocated to the control intervention will receive treatment as usual (primary care visits, antidepressant medication if indicated), weekly mood monitoring, and guided self-help (Managing Stress on the Farm), representing the standard first-line treatment option for people with depression. An automated email directing participants to complete their mood rating will be sent weekly along with a reminder SMS sent to their mobile phone. The workbook provides tailored information, practical activities, and links to further resources (including what to do in a crisis) to support the mental health of members of the farming community. A weekly ‘check-in’ addresses some of the attentional issues related to a self-help workbook control intervention.

An attentional control—to address the non-specific effects of the experimental intervention—was discussed as an alternative comparator intervention. Although there are methodological advantages, we did not consider an attentional control to be consistent with our ethical obligation to ensure that participants were receiving standard of care (guided self-help). That said, we acknowledge the potential that this may overestimate the acceptability of the Back-on-Track intervention.

### 2.7. Outcomes

Outcome measures will be administered at baseline (week 0) and at weeks 10 and 26 follow-up. The primary outcome (to be used in a definitive trial) is depressive symptoms determined by change in PHQ-9 scores [24]. The PHQ-9 is a self-administered measure of depression symptoms that has been extensively used in clinical trials of psychological treatment.

Secondary outcomes (proposed) are as follows:

1. Enhanced work performance—determined using the Work Productivity and Activity Impairment questionnaire [25], a six-item validated instrument to measure impairments in both paid work and unpaid work.
2. Reduced loneliness—determined using the University of California, Los Angeles (UCLA) loneliness scale [26], a 20-item validated scale designed to measure the participant’s subjective feelings of loneliness as well as feelings of social isolation.
3. Enhanced overall wellbeing—determined using the WHO-5 Wellbeing index [27], a short self-reported measure of current mental wellbeing.
4. Improved overall quality of life—determined using the AQoL-4D [28], a validated, health-related multi-attribute utility instrument.

Outcome measures are estimated to take a total of 20 min to complete. For noting, we will not conduct or report any form of statistical analysis to determine the efficacy of the Back-on-Track intervention.

### 2.8. Treatment Fidelity

Treatment fidelity will be determined using the Behavioural Activation fidelity assessment—found to be reliable, feasible, and acceptable in previous studies [20]. A random sample (approximately 20, 10%) of all sessions conducted during the trial will be recorded using Zoom. At the end of the trial, they will be blind rated by an independent (not part of the research group) clinician experienced in Behavioural Activation. A 10-percent sample, for the purposes of fidelity checking, is typical in trials of novel psychological treatments. Peer workers will need to be rated as three (moderately) or higher against each of the 10 items of the Behavioural Activation fidelity assessment (a total score ≥ 30). Fidelity ratings will not be completed until after the treatment has concluded; consequently, it will not be possible to provide feedback to peer workers whilst treatment is ongoing, which is an acknowledge limitation of our fidelity-monitoring procedure.

Providing consent for audio recording of sessions will be part of the participation consent process, but not compulsory. Participants will have the option to indicate that they do not want sessions to be recorded.

### 2.9. Economic Data

Costs—including the cost of training and delivering the intervention (Back-on-Track) and the cost of the control (Managing Stress on the Farm self-help workbook)—will be calculated. Identified costs for the Back-on-Track intervention will include peer worker training, training materials, peer supervision (e.g., clinical psychologist time), staff co-ordination, administrative overheads, and delivery time per participant. For the control group, identified costs will include components of treatment as usual (e.g., GP visits and antidepressant prescriptions where indicated), the provision of the guided self-help materials, and automated weekly mood monitoring.

Additionally, healthcare service use data will be collected throughout the trial period including Medicare Benefits Schedule (MBS—primary care attendance), Pharmaceutical Benefits Scheme (PBS—prescribed medications via Services Australia), and the Victorian Admitted Episodes Dataset (VAED) via Victorian Agency for Health Information (VAHI) for emergency department and inpatient hospitalisation data. Participant-level data on health service utilisation will be linked and analysed to quantify any differences in service use between the intervention and control groups, capturing both physical and mental health-related encounters.

### 2.10. Demographic and Clinical Information

Basic demographic information (age, sex) will be collected from peer workers.

The following information will be collected from farming community participants:

- Demographic—age, sex, postcode, residential status, employment status, indigenous status, culturally and linguistically diverse status, occupation in the farming community.
- Clinical—self-reported long-term health conditions, previous depression treatments.

### 2.11. Sample Size

The intended sample size is 40 participants (approximately 20 in the intervention supported by 10 peer workers and 20 in the control groups). Sample size calculations are not appropriate for feasibility trials, and a sample of 40 is generally considered adequate to establish that a definitive trial is feasible [29].

### 2.12. Recruitment

#### 2.12.1. Procedures for Recruiting People with Depression

Based on figures provided by Agriculture Victoria, we estimate that 69,000 people are employed in agricultural production in Victoria. Trials of Behavioural Activation are typically able to recruit around one in three people that are asked to participate [30]. We anticipate that around a third of people (approximately 23,000) in the farming community will meet our inclusion criteria and predict that around 200 potential participants will express an interest in participating in the trial. Recruitment will be monitored at our weekly trial management groups and, if fewer people than expected indicate interest, we will revise our recruitment strategy in consultation with our Community Reference Group.

#### 2.12.2. Recruitment Procedures

Advertising and promotion of the trial will be through existing National Centre for Farmer Health communication networks (social media, website, and monthly e-newsletter). Government, industry, community partners, and rural media contacts will be requested to promote the trial through their communications networks using recruitment material informed by a community consultation process. This approach should enable us to reach most members of the farming community. The number of contacts we obtain from this approach will inform the design of a definitive trial.

We will monitor recruitment rates at our weekly trial management team meetings. If we are not likely to recruit to time and target, we consider early revisions to our recruitment strategy through discussions with investigators and consultation with our community advisory group.

#### 2.12.3. Pre-Screening and Consent Procedures

Members of the farming community interested in participating in the trial will be asked to submit an expression of interest by telephone, email, or online. The researcher will contact potential participants by phone to discuss the trial in detail and to obtain verbal consent to complete screening. At the start of the screening interview, participants will be asked to complete the PHQ-9. Researchers will total the score and feedback to participants.

PHQ-9 cut-off scores from Kroenke et al. [24] will be used. Potential participants scoring below five or over 19 on the PHQ-9 will be notified that they do not meet trial inclusion criteria and are not eligible to take part in the trial. Ineligible participants will be sent a copy of the Managing Stress on the Farm self-help workbook. Ineligible participants scoring over 19 (indicative of severe depression) will be informed by the researcher that their score suggests that they may be experiencing severe depression and will be asked if they are currently receiving any psychological or pharmacological treatment for their mental ill-health. If they are, the researcher will advise the individual to contact their mental health clinician as soon as possible. In instances where the individual does not have a mental health clinician, they will be advised to contact a health professional. The researcher will explain that, due to the severity of their psychological symptoms, they do not meet trial inclusion criteria.

Potential participants that meet trial inclusion criteria will be given a detailed verbal explanation of the trial procedures and informed that they will receive an email containing a written Participant Information and Consent Form (PICF) to review and sign. If they are unable to receive the PICF via email, a paper copy will be mailed to them. If no signed PICF (electronic or paper copy) is received after 7 days, the potential participant will be followed up by phone, asking if they still wish to take part in the research.

During the consent process, participants will be informed that the information they provide will be kept strictly confidential except in circumstances where they indicate that they may harm themselves or other people. The risk of harm to self will be determined from discussion with participants.

Participants will be informed that, if they feel that they are at imminent risk of harm to self or others, they must tell the researcher, who will then advise them to contact their GP, the Emergency Department, or emergency services as appropriate.

#### 2.12.4. Inclusion of Young People Aged 15–17 Years

The prevalence of depression in young people aged 15–17 year and their early involvement in farming communities justifies their inclusion in this trial. Written consent will be sought from both young people and their parent or guardian. Including young people in the trial is informed by evidence that around a quarter of the agricultural workforce in 2021 were aged 15 to 35 years [31].

We acknowledge that depression in young people can present somewhat differently to adults [32]. For example, they may express more anger, irritability, oversensitivity to criticism or rejection, selective withdrawal, and unexplained aches and pains. However, the PHQ-9 is still considered an appropriate screening tool for adolescents [33]. For example, in a validation study involving 2235 students aged 12–18, years of age, Fonseca-Pedro [33] showed the PHQ-9 was a brief, easy, and valid screening measure in adolescents.

#### 2.12.5. Recruitment and Informed Consent Process (Peer Workers)

Peer worker positions will be advertised publicly through established National Centre for Farmer Health networks including social media and e-newsletters. Potential peer workers may also be identified through expressions of interest or via the community consultation process. Potential peer workers will apply for casual positions and will be employed by the Western District Health Service. Information about the position will include details of the research and the requirement for peer workers to participate. Applicants that meet the essential selection criteria will be invited for interview. The panel will be chaired by the chief investigator and will include two other members of the research team, including at least one lived experience researcher (with experience of living and working in a farming community). During the interview, the chair will provide a verbal description of the trial and indicate that consenting to participate in the research is a requirement of the role. Successful candidates will be offered a casual contract of employment and required to provide written informed consent to participate in the trial.

#### 2.12.6. Baseline Measures

Following consent, participants will complete week 0 (baseline) measures entered and stored on a secure password protected platform, REDCap (Research Electronic Data Capture)—a secure, web-based software platform designed to support data capture for research studies [34]. The platform is approved for use in clinical trials by both Deakin and La Trobe Universities. Participants unable to complete measures independently online will be supported by a researcher who will ask participants questions verbatim and enter responses into REDCap on their behalf. On completion of baseline measures, eligibility checklists will be finalised, and the participant will be randomised in REDCap.

Automated reminders (email and text message) will be sent 48 h and 5 days post-submission-of-consent if baseline measures are not completed. A phone call and a final reminder will be actioned on day 7. If participants fail to complete trial measures by the end of day 7, they will be informed that they have been withdrawn from the trial.

### 2.13. Allocation

Randomisation will be undertaken using an online randomisation service—Sealed Envelope Ltd London, UK [34]—and managed by the La Trobe University Clinical Trials Platform to ensure the research team remain blinded to group allocation. Randomisation will be by random permuted blocks with a 1:1 ratio.

The Clinical Trials Platform will contact the peer worker by phone and inform them of the allocation of a new trial participant and ask if there is any known conflict of interest with the allocated participant. If there is no conflict of interest, the peer worker will be provided with allocated participant details (name, telephone number, and email address) and will call the participant to introduce themselves and arrange the first session.

If there is a conflict of interest identified (either by the peer worker or participant), the participant will be reallocated to a different peer worker. Participants allocated to the control group will be contacted by phone by the Clinical Trials Platform to inform them of the allocation, emailed an electronic copy, and sent a hard copy of the Managing Stress on the Farm workbook in the post.

### 2.14. Blinding (Masking)

This is a single-blind trial. All research team members will be masked to group allocation with the exception of the Clinical Trials Platform team, who will need visibility of allocation to provide contact details to peer workers and mail resources to participants in the control group. Peer workers will be delivering the intervention and as such will not be masked.

As part of the consent process, a full explanation of the trial design will be given to participants including a description of the Back-on-Track (experimental) and Managing Stress on the Farm self-help workbook (control) interventions. Consequently, participants will not be blind to group allocation (an open-label trial). Awareness of group allocation can impact how participants respond to the intervention they receive. Participants who know they have been allocated to Back-on-Track may have more favourable expectations than those allocated to the Managing Stress on the Farm group, who may feel disappointed. Participants in the trial may assume that Back-on-Track will be better than Managing Stress on the Farm. Telling participants which intervention they will receive may also influence how participants complete outcome assessments. It is also possible that knowledge of treatment allocation may also impact participant compliance and dropout from the study. It will be important when interpreting the outcomes of this feasibility trial to acknowledge the context within which the research was conducted.

#### 2.14.1. Blinding Mechanism

Assessments will be completed online by participants or via videoconference with the assistance of a researcher who will be masked to the treatment allocation. Researcher training will include the importance of adherence to the trial protocol and maintaining blinding. For example, emphasising the importance of not asking trial participants if they received the Back-on-Track or Managing Stress on the Farm intervention (at follow-up assessments).

To test the effectiveness of allocation concealment procedures trial, researchers will be asked at the end of the trial which group they thought participants were allocated to. We will then test if the prediction was greater than chance.

#### 2.14.2. Emergency Unblinding

In exceptional circumstances—for example, a sequence of adverse or serious adverse events that seem to be related to the intervention—blinding will need to be broken. In such circumstances, the trial researcher will contact the chief investigator to convene an emergency trial management group meeting to review the events and consider if unblinding is necessary.

The breaking of blinding would not necessarily stop the trial but would need to be clearly reported in any publication or reports of the trial findings.

## 3. Data Collection

### 3.1. Trial Procedures and Evaluations

Participants will be asked to complete the electronic Case Record Form (eCRF) at baseline (week 0), end of treatment (week 10), and week 26 via REDCap or with the help of a trial researcher, who will enter the information into REDCap on the participants’ behalf. Table 1 shows the notification schedule for the electronic case record form process. The participants will be considered to have withdrawn from the trial if they fail to complete outcome measures within the required time frame of 14 days or do not respond to two sequential messages from their peer worker (phone or text).

### 3.2. Data Management

The electronic case record form will be developed by the Clinical Trials Platform. To promote data quality, the electronic case record form will be designed to minimise errors in data entry, e.g., not permitting the entry of impossible values (e.g., age of 999 years). Wherever possible, we will use drop-down menus or tick boxes that are easy for participants to accurately complete. Survey logic will be used to ensure that participants are not having to skip over or answer irrelevant questions. Participants will need to answer all compulsory questions before submission, eliminating the risk of missing data.

### 3.3. Statistical Methods

Descriptive statistics (predominantly number and proportion) will be used to summarise the feasibility outcomes from our trial. We will report means and standard deviations for the five outcome measures at the three trial time-points but will not undertake any statistical analysis.

## 4. Economic Evaluation

The economic evaluation reporting will adhere to the Consolidated Health Economic Evaluation Reporting Standards (CHEERS) statement [35]. A cost-consequence analysis will explore the costs associated with the programme and report these alongside potential benefits and outcomes. A cost-consequence analysis (CCA) is a form of economic evaluation where costs and outcomes are listed separately rather than combined into a single metric (such as a cost-effectiveness ratio). This approach allows decision-makers to consider a range of impacts and make context-specific judgements about value and trade-offs. The analysis will be conducted from the ‘funder perspective’, allowing health care organisations to consider implementation cost for this and similar programmes.

Consequences will include the primary clinical outcome of depressive symptoms (measured by change in PHQ-9), along with secondary outcomes such as enhanced work performance (Work Productivity and Activity Impairment questionnaire), reduced loneliness (UCLA Loneliness Scale), improved wellbeing (WHO-5), and health-related quality of life (AQoL-4D). Patterns of health service use (e.g., GP visits, hospital presentations, mental health service contacts) will also be reported as a key outcome, reflecting potential shifts in demand associated with the intervention. These outcomes capture a broad range of potential programme benefits and align with the lived experience goals of the target population.

Costs and outcomes will not be aggregated into a single cost-effectiveness ratio, in line with the cost-consequence approach, but will be presented in a disaggregated format to support decision making. Sensitivity analyses will be conducted to test assumptions around intervention delivery costs and variability in health service use. In addition, outcomes such as the AQoL-4D will provide utility weights that may be used in future cost-utility analyses.

Findings from this feasibility-phase CCA will directly inform the design of a future definitive trial, including refinement of data collection tools, choice of primary outcome, and economic parameters. The results will also be used to support the development of a longer-term economic model (e.g., decision tree or Markov model) to estimate cost-effectiveness over time. This model will enable estimation of cost-per-quality-adjusted life years (QALY) gained and provide critical information for funders and policymakers considering broader implementation.

### 4.1. Process Evaluation

#### 4.1.1. Interviews with Peer Workers and Trial Participants

Peer workers interviews will be conducted at the end of the trial once all Back-on-Track sessions are complete. Interviews will assess the acceptability, feasibility, barriers, and facilitators to delivering the Back-on-Track intervention.

Interviews with 10 participants (five from each group) will be conducted to assess the acceptability, feasibility, barriers, and facilitators to engagement with the trial. All interviews will be conducted as soon as possible after intervention group participants have completed their final Back-on-Track session and after control group participants have completed week 10 assessment measures.

Interviews will be conducted by research team members following a semi-structured interview schedule and transcribed using a transcription service. We will use the framework method, following [36], to analyse the interviews.

##### Post-Trial Survey of Participants

All trial participants (intervention and control) will be sent (via email or post based on preference) a post-trial survey about their involvement in the research. Questions drawn from the study participant feedback questionnaire toolkit [37] will explore the adequacy of information about the trial, experiences of participating, motivation to take part in research, understanding of randomisation processes, and perceived value of the study.

#### 4.1.2. Data Monitoring

##### Formal Committee

Behavioural Activation has been established in previous studies as a safe treatment for depression with a low risk of adverse events; therefore, the trial does not require a formal Data and Safety Monitoring Board (DSMB). Trial progress will be monitored through the Trial Steering Committee that is independently chaired.

### 4.2. Safety Monitoring

Participants will complete trial measures at three time-points: baseline (week 0), end of treatment (week 10), and follow-up (week 26). During the assessments, participants will be required to self-report any medical occurrences, GP visits, hospital admissions, or if they have been sick or unwell (physically or mentally) during the trial period. They will also be asked to report any suicidal thoughts or suicide attempts that have occurred.

An SMS will be sent to participants and peer workers every 2 weeks using REDCap (via TWILIO—a third-party web service), asking them to report any adverse events that occurred. The adverse event reporting period spans from the time of consent through to the completion of week-26 assessments. Adverse events will be graded according to the Common Terminology Criteria for Adverse Events (CTCAE) Version 5.0 [38].

Causality of adverse events will be assessed by the principal investigator to determine if there is a possible link with the trial intervention. The trial will cease immediately if there are any suicide attempts or fatalities that are plausibly linked to either the trial intervention or trial procedures. Adverse events will be reported to the trial sponsor and the relevant human research ethics committee.

### 4.3. Community Reference Group (CRG)

A Community Reference Group (comprising community members, service providers, and key stakeholders from the three trial communities) will inform strategies for the recruitment and engagement of peer workers and participants. The Community Reference Group will meet monthly for 6 months to provide input on the tailoring of training materials to accurately reflect the farming context, then as required during the trial period to problem solve emerging issues, e.g., difficulties in recruiting peer workers, slower-than-expected participant recruitment rates.

### 4.4. Ethics and Dissemination

#### 4.4.1. Research Ethics Approval

The trial will be conducted in accordance with Good Clinical Practice Guidelines (ICH GCP, https://database.ich.org/sites/default/files/E6_R2_Addendum.pdf accessed on 20 January 2026). The trial was prospectively registered with ACTRN on 23 April 2024. The St Vincent’s Hospital Human Research Ethics Committee (HREC) (Ref: 070/24) reviewed and approved the trial protocol. Trial documents and a copy of the ethics committee approval letter have been reviewed and approved by the La Trobe University HREC (Ref: HREC 070/24), University of South Australia HREC (Ref 206358), Deakin University HREC (Ref 2024-203), and Edith Cowan University HREC (Ref 2024-05841-JONES).

#### 4.4.2. Distress Management Protocol

A stepped approach will be adopted to manage people experiencing distress or thoughts or plans for self-harm during the trial. All members of the research team and peer workers will receive training around the distress protocol. The peer worker programme delivery manual will include a flowchart outlining the distress protocol for ongoing referral. The participant workbook will include a flow chart outlining the process for participants to seek mental health support should distress be experienced outside of meetings with their peer worker, including direction to local supports where available. Participants will also be offered the opportunity to speak with a psychologist if they withdraw from the trial due to heightened distress.

Control group participants will also be sent weekly mood-monitoring checks. The Clinical Trials Platform will be notified if there is a decline in the mood-monitoring score for 2 consecutive weeks or if the score is less than or equal to four for 2 consecutive weeks. The Clinical Trials Platform will notify the trial psychologist, who will contact the participant to assess their mental health and make any necessary referrals.

Researchers and peer workers will follow the process as above when working with young people aged 15 to 17 years. Additionally, when young people express concern about harming themselves, parents or guardians will be informed about the identified risk.

### 4.5. Confidentiality

Trial data will be directly entered into the electronic case record form. There will be a physical log of participant group allocation (participant names, group allocation, and trial number) that will be securely stored at La Trobe University.

Dissemination Policy: The results of the trial will be reported in an open-access peer-reviewed journal. A summary of findings of the trial will also be published on the National Centre for Farmer Health website.

## 5. Conclusions

This feasibility trial will contribute to evidence informing the future design and implementation of mental health support models in Australia’s farming communities, improving access to appropriate and accessible mental health support in an identified at-risk population.

## Figures and Tables

**Table 1:** Electronic Case Record Form notification schedule.

Day	Content	Format
0	Request to complete outcome measures	Survey link in email notification, SMS reminder
3	Reminder to complete outcome measures (if not completed)	Survey link in email notification, SMS reminder
6	Reminder to complete outcome measures (if not completed)	Survey link in email notification, SMS reminder
8	Notification to researcher to follow up participant by phone if outcome measures not complete	Email notification to researcher

## Data Availability

The original contributions presented in this study are included in the article. Further inquiries can be directed to the corresponding author.

## Consumer Involvement Statement

Consumer involvement was central to the design of this trial. Farmers, the agricultural industry, and healthcare representatives and rural community stakeholders were consulted throughout the co-design and development of the trial. Community consultation workshops informed local stakeholder engagement and recruitment methods. A Community Reference Group and Trial Steering Committee guided the tailoring of training and recruitment materials, delivery methods, and participant recruitment processes to reflect the farming context. This collaborative model ensures that the intervention is both evidence-based and grounded in the lived experiences of the farming community, enhancing its feasibility and long-term sustainable impact.

## Abbreviation

WDHS	Western District Health Service
